# Dynamic changes in the cell membrane on three dimensional low coherent quantitative phase microscopy (3D LC-QPM) after treatment with the near infrared photoimmunotherapy

**DOI:** 10.18632/oncotarget.22223

**Published:** 2017-11-01

**Authors:** Fusa Ogata, Tadanobu Nagaya, Shuhei Okuyama, Yasuhiro Maruoka, Peter L. Choyke, Toyohiko Yamauchi, Hisataka Kobayashi

**Affiliations:** ^1^ Molecular Imaging Program, Center for Cancer Research, National Cancer Institute, National Institutes of Health, Bethesda, Maryland 20892, United States of America; ^2^ Central Research Laboratory, Hamamatsu Photonics K.K., Hamamatsu 434-8601, Japan

**Keywords:** near Infrared photoimmunotherapy, cell membrane damage, live cell imaging, immunogenic cell death

## Abstract

Near infrared photoimmunotherapy (NIR-PIT) is a newly developed cancer therapy that relies on the binding of a near-infrared antibody photoabsorber conjugate (APC) to a cancer cell. Subsequent exposure to NIR light selectively induces rapid necrotic cell death on target-expressing cells with minimal off-target effects. When treated with NIR-PIT, targeted cells become swollen, develop blebs and burst within minutes of light exposure. Detailed spatial and temporal morphological changes of the cellular membrane of targeted cells treated with NIR-PIT have not been fully explored with state-of-the-art microscopic methods. In this study, we investigated the morphologic and kinetic effects of PIT on two types of cells, a spindle-shaped 3T3/Her cell and a spheric-shaped MDA-MB468 cell, after NIR-PIT using three-dimensional low-coherent quantitative phase microscopy (3D LC-QPM). Adhesive cells treated with NIR-PIT demonstrated region-specific cell membrane rupture occurring first on the distal free edge of the cell near the site of adhesion, in a process that was independent of cell shape. The results show that the peripheral portions of the cell membrane near the site of adhesion are particularly vulnerable to the effects of NIR-PIT, likely because these sites exhibit higher baseline surface tension.

## INTRODUCTION

Near infrared photoimmunotherapy (NIR-PIT) is a newly developed cancer therapy in which a monoclonal antibody is conjugated to a near-infrared photoabsorber, IRDye700DX (IR700), which is a silica-phthalocyanine derivative [1]. After the antibody-photoabsorber conjugate (APC) is administered and specifically binds to the cancer cell membrane expressing the cognate antigen, NIR light activates the APC resulting in highly selective cell death of the cancer cells with minimal effects on surrounding normal cells. Within minutes of NIR light exposure, the targeted cells become swollen, bleb and rupture [2, 3]. The process of cancer cell death following NIR-PIT occurs within several minutes of NIR exposure.

The two major processes of cell death are necrosis and apoptosis, which can occur independently and simultaneously [4]. The rapid and irreversible damage of the cell membrane function induced by NIR-PIT is predominantly necrotic rather than apoptotic which differentiates it from most other cancer therapies [5, 6] The rapidity with which cell contents are disgorged into the extracellular microenvironment leads to activation of the immune system augmenting tumor response [7, 8]. Thus, NIR-PIT is a novel therapy that rapidly and efficiently induces immunogenic cell death (ICD) via cellular necrosis.

ICD is a concept that has emerged in the past decade [9, 10]. Rapidly induced necrosis leads to the release of cell surface fragments as well as intracellular contents. Inflammatory and immune cells including mature dendritic cells (DCs) in the microenvironment of primary cancer lesions are activated. Ultimately, this unleashes a potent anticancer immunity in which CD8^+^ cytotoxic T lymphocytes (CTLs) are key effector cells [11]. NIR-PIT has been reported to induce mature DCs resulting in the infiltration of CTLs [7, 8] leading to killing of the targeted cancer cells [12, 13].

The exact mechanism by which the cell membrane is damaged, thus initiating ICD, is still a matter of debate. It is a reasonable assumption that the primary mechanical force induced by NIR-PIT is quite strong, sufficient to weaken the membrane to its breaking point. However, the spatial and temporary changes immediately after NIR-PIT remain unclear due to the difficulty of evaluating rapidly occurring changes in the cell membrane.

Three dimensional low coherent-quantitative phase microscopy (3D LC-QPM) is a reflection-type interference microscope that uses a low-coherent light source to serially obtain depth-resolved images of the cell membrane at sub-micrometer resolution in unmodified cells without labeling [14, 15]. This technique is based on interferometry between the sample light reflected from the cell surface and the reference beam and can evaluate the full field surface topography of cultured cells and intrinsic membrane motion with a sensitivity of tens of nanometers. 3D LC-QPM could be a sensitive method to detect three-dimensional dynamic changes in the cell membrane induced by NIR-PIT. Hence, in this study we employ 3D LC-QPM to investigate the membrane dynamics after NIR-PIT in two types of cells, spindle 3T3/Her cells and spherical MDA-MB468 cells.

## RESULTS

### Cancer cells rapidly enlarge after NIR-PIT

**Figure 1:**
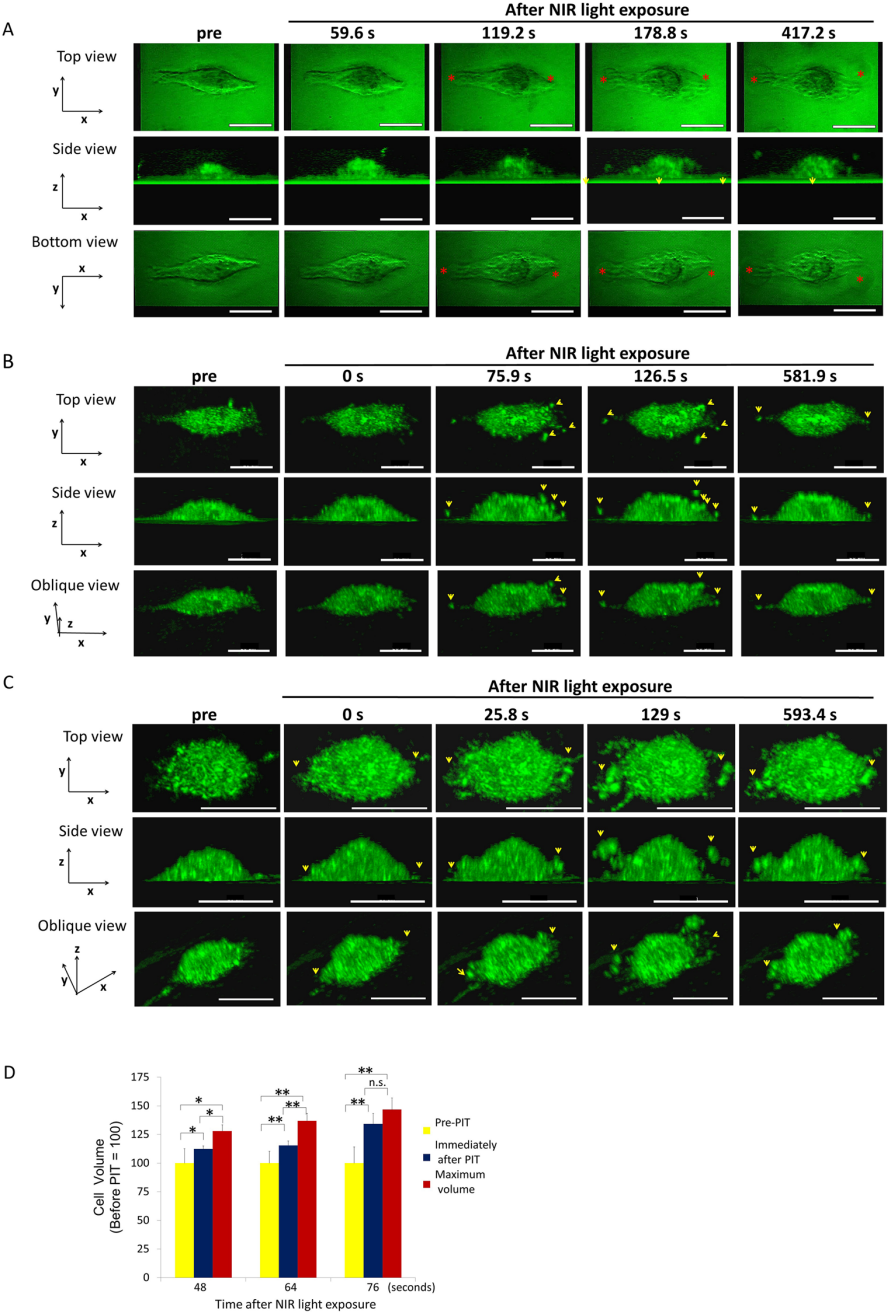
3T3/Her cells increased in volume and ruptured after NIR-PIT Representative images from 3D-LC QPM imaging depict morphological dynamics in three regimens of NIR light exposure time, 48 sec **(A)**, 64 sec **(B)** and 76 sec **(C)**. The cell in (A) initially swelled, formed blebs and ruptured. The cell in (B) and the cell in (C) ruptured without antecedent bleb formation and in (C) had already burst by the end of NIR light exposure. Red asterisk indicates a bleb. Yellow arrowhead indicates a flying fragment. See also Supplementary Videos 1-3 which are side views of cells (A, B, and C). **(D)** Comparison of cell volumes before and immediately after NIR light exposure and maximum volume. The cells showed significant cell volume increases after NIR-PIT. Longer NIR light exposures (76 sec), caused more damage and there was no significant difference between the volume immediately after NIR light exposure and the maximum volume. Data are means ± SE. n = 12 in 46 sec and 64 sec. n =10 in 76 sec. ^*^P < 0.05, ^**^P < 0.01, ^***^P < 0.005 versus the other group. n.s. indicates no significant difference. s indicates second or seconds.

**Figure 2:**
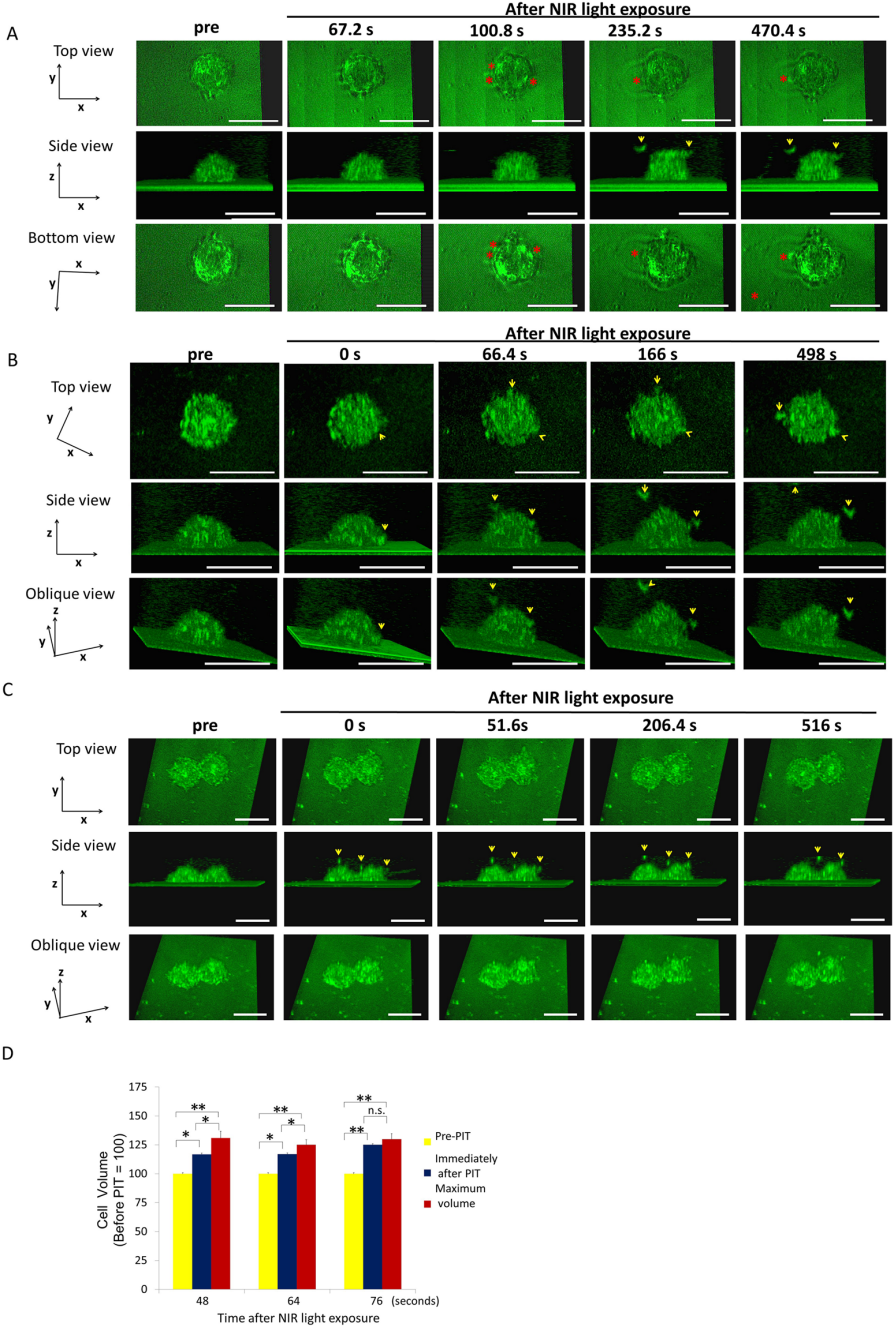
MDA-MB468 cells increased in volume and then ruptured after NIR-PIT Representative images from 3D-LC QPM imaging depict morphological dynamics using three regimens of NIR light exposure time, 48 sec **(A)**, 64 sec **(B)** and 76 sec **(C)**. The cell in (A) swelled followed by bleb formation and then ruptured. The cell in (B) and the cell in (C) ruptured without antecedent bleb formation; it had already burst at the conclusion of the NIR light exposure. Red asterisk indicates a bleb. Yellow arrowhead indicates a flying fragment. **(D)** Comparison of cell volume before and immediately after NIR light exposure and maximum volume. Treated cells were all dramatically increased in volume. The cells significantly increased in cell volume compared to baseline after NIR-PIT. The cells exposed to longer NIR light, (e.g. 76 sec), didn’t show significant cell volume increases, which suggested that the damage was complete by the end of the light exposure. Data are means ± SE. n = 12 in 46 sec and 64 sec. n =10 in 76 sec. ^*^P < 0.05, ^**^P < 0.01, ^***^P < 0.005 versus the other group. n.s. indicates no significant difference. s indicates second or seconds.

The dynamic 3D LC-QPM imaging showed that 3T3/Her cells and MDA-MB468 cells increased in volume and changed in shape following NIR light exposure (Figures 1 and 2). Shape changes are described in more detail below.

### 3T3/Her cell changes after NIR light exposure

3T3/Her cells are elongated and flat with wide fan-like lamellas. The cells exposed for 48 seconds (sec) of NIR light swelled and, simultaneously formed a few blebs which grew larger and accreted into larger blebs before rupturing (Figure 1A). A maximum cell volume (up to 30% greater than baseline) occurred before the cell ruptured and this occurred at a mean of 154.67 ± 8.36 sec after the NIR light exposure (Figure 1D). In cells exposed to NIR light for 64 sec, rupture had already occurred by the time of imaging in 8 out of 24 cells (Figure 1B). The cellular volume showed a 15% increase immediately after exposure compared with the initial volume and the maximum cell volume reached 20% > baseline prior to rupture (Figure 1D). Cells ruptured at a mean of 49.61 ± 11.25 sec after the NIR light exposure. In cells exposed to NIR light for 76 sec, rupture had already occurred by the time of imaging in 10 out of 12 cells (Figure 1C) but in the 2 unruptured cells occurred when the mean volume increase was 34% over baseline (Figure 1D). Two of the 12 cells had actually decreased in volume by the time of imaging due to prior rupture of the cells during NIR exposure. As a result there was no significant difference between the volume immediately after the NIR light exposure and the maximum volume (Figure 1D). The cells exposed for more than 100 sec NIR light were all ruptured during the light exposure.

### MDA-MB468 cell changes after NIR light exposure

MDA-MB468 cells are round with narrow lamellas. MDA-MB468 cells exposed to 48 sec of NIR light reached a maximum volume of 33% greater than baseline before blebs formed (Figure 2A, 2D). The blebs formed at the protruding lamellae and then expanded rapidly and locally fused into a single large bleb. Eventually, the cell membrane ruptured at a mean of 447.02 ± 52.28 sec from light exposure. The cells exposed to NIR light for 64s (Figure 2B) demonstrated maximum volume increases of 41% greater than baseline prior to rupture at a mean of 77.52 ± 10.79 sec after NIR light exposure (Figure 2D). The cells exposed to NIR light for 76 sec NIR light exposure had already ruptured during the NIR light exposure (Figure 2C) and there was no significant volume increase (Figure 2D).

### Ruptures occurred at the periphery of cells after NIR-PIT

**Figure 3:**
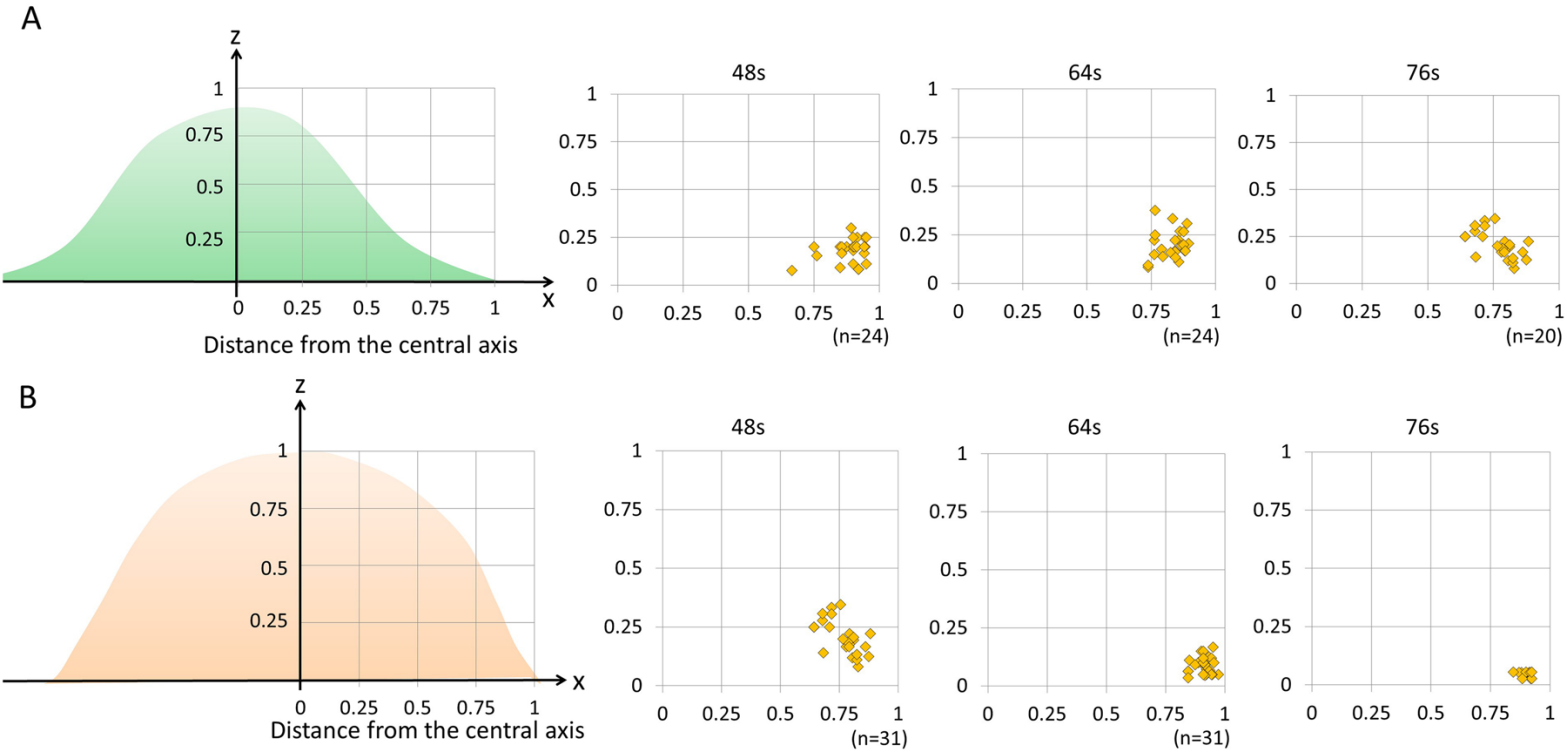
The distribution of blebbing and cell rupture on the cell membrane vs. distance from the thickest part of the cell 3T3/Her cells (n = 24 in 48 sec and 64 sec, n = 20 in 76 sec) **(A)** and MDA-MB468 cells (n = 18 in 48 sec, n = 31 in 64 sec, n = 10 in 76 sec) **(B)**. Both cell types showed that damage occurred primarily in the outer one fourth of the cell membrane close to where the free margin of the cell joins the adherent cell membrane. s indicates seconds.

The location of cell rupture was similar in both cell types. 3T3/Her cells and MDA-MB468 cells both burst preferentially on the peripheral free edge of the cell near its contact adhesion to the slide surface (Figure 3). In 3T3/Her cells both sides of the long axis of the cell were deformed and burst. In MDA-MB468 cells bleb formation was irregularly observed. The blebs were consistently observed near the interface with the cell adherent to the slide surface, not more than 0.84 µm height above the slide surface which is one twentieth to one twenty fifth of the cell height. As a result the view from the bottom of the cell was useful to observe the blebs (Figures 1A and 2A).

## DISCUSSION

NIR-PIT is a highly selective and effective cancer treatment for tumors [16–19]. A Phase I/II trial of NIR-PIT in patients with recurrent head and neck cancer (ClinicalTrials.gov Identifier: NCT02422979) is underway. The trial has recently advanced to Phase II. Preliminary results suggest NIR-PIT is safe and effective (Merrill Biel, MD, personal communication). Several reports have demonstrated dynamic changes in targeted cells after NIR-PIT using conventional microscopy. For instance, filopodia rapidly disappear [3] and cellular swelling and rupture [2, 7, 8] are observed and appear irreversible. However, a detailed mechanism by which rupture of the membrane occurs is not known. To gain insight, we investigated the membrane damage using a live cell imaging system.

Conventional microscopic imaging techniques of live cell membranes using fluorescently labeled surface proteins is the classic method of assessing cell membrane disruption. However, imaging time is limited by loss of fluorescence signal due to photobleaching [20] and the biology may be influenced by phototoxic effects of excited fluorophores, which themselves produce reactive oxygen species (ROS) and can eventually lead to cell death [21–23]. 3D LC-QPM is a new, non-invasive imaging method for evaluating cell membrane dynamics in real time. It is very sensitive to cell membrane disruptions due to the accuracy with which it can detect small changes in cell volume. At the same time no toxic fluorophore labels are needed. Our results demonstrate that 3D LC-QPM localizes the site of initial cell membrane blebbing and rupture following NIR-PIT in both 3T3/Her cells and MDA-MB468 cells. Since it does not use fluorescent labels, 3D LC-QPM provides a useful and non-invasive method of assessing shape and rupture of cell membranes induced by NIR-PIT.

Blebbing is not necessarily indicative of cell necrosis and is commonly observed during apoptosis [24], cell migration [25], cytokinesis [26] and cell membrane repair against physical stress [26–28]. Some studies have reported that blebs grow in regions where membrane–cortex attachment is weaker [29, 30] or where the hydrostatic pressure inside the cell is locally higher [26]. In this study we observed blebs prior to cell membrane rupture after NIR light exposure in cells incubated with appropriate APCs. The blebs enlarged, coalesced and then ruptured leading to stabilization or even decreases in cell volume. Blebs can be seen with membrane repair mechanisms but in that case the blebs form after rupture, not before. Blebbing was visible when the NIR light exposure was 48-64 sec long. Blebbing was not seen in cells exposed to NIR light for >64 sec perhaps because it had already occurred by the time imaging began or perhaps because there was no blebbing before rupture with longer exposure times due to the faster kinetics of membrane damage.

NIR-PIT treated cells displayed a variety of morphologies which were light dose dependent. However, intriguingly, the points of rupture were uniformly in the peripheral outer one-fourth of the cells and not seen in the more central portions of the cell (Figure 3). Since both APCs are homogeneously distributed on the cell membrane of respective cells (Supplementary Figures 1 and 2), NIR-PIT-induced membrane damage should be uniform. However, the most blebbing and cell rupture was seen near the interface between the free margin of the cell and the adhered margin on the slide. These results raise the possibility that this peripheral region is more vulnerable to NIR-PIT. This region of the cell has the highest surface tension because the adhered part of the cell or tapering part of the spindle cell has the greatest membrane tension. Additionally, the central portion contains radially oriented thick filaments named microtubules while the peripheral membrane is supported by short linear actin bundles and a mesh-like actin network in lamellipodial veils. These appear to be more fragile and susceptible to cell membrane damage. The cells are easily cultured in a single monolayer culture dish which is sufficient to observe initial changes on the cell membrane by NIR-PIT because NIR-PIT tends to preferentially kill superficial cancer cells adjacent to vessels, at least initially. However, for further analysis to observe NIR-PIT-induced tumor shrinkage caused by successive cell membrane damages in deeper layers, the three-dimensional culture and other microscope system including 2-photon microscopy would be needed [31–34].

In conclusion, 3D LC-QPM is a useful tool for studying real-time imaging of the cell membrane after NIR-PIT. It helps identify the outer fourth of the cell membrane as most vulnerable to the effects of NIR-PIT as this is the site of initial blebbing and cell rupture. This suggests that the peripheral cell membrane in adhesive cells may be subject to higher structural and mechanical forces. Naturally, such forces are not necessarily in play *in vivo* as cells may not be adherent, but sites of adhesion are likely to be the most vulnerable. While the molecular mechanism of cell membrane damage remains under investigation these results provide new insights into the ongoing analysis of the therapeutic mechanisms of NIR-PIT.

## MATERIALS AND METHODS

### Cells

HER2-expressing 3T3/Her cells, which are cultured human fibroblast cells, and HER1-expressing MDA-MB468 cells, which are a cultured human breast cancer cell line, were used as target cells for NIR-PIT. They were grown in RPMI 1640 supplemented with 10% fetal bovine serum and 1% penicillin/streptomycin in tissue culture flasks in a humidified incubator in 5% carbon dioxide at 37 °C.

### Synthesis of IR700-conjugated trastuzumab or panitumumab

Trastuzumab or panitumumab (1 mg, 6.8 nmol) was incubated with IR700 (66.8 μg, 34.2 nmol) in 0.1 mol/L Na_2_HPO_4_ (pH 8.6) at room temperature for 1 hour. The mixture was purified with a Sephadex G25 column (PD-10; GE Healthcare, Waukesha, WI). The protein concentration was determined with Coomassie Plus protein assay kit (Thermo Fisher Scientific Inc., Rockford, IL) by measuring the spectroscopic absorption at 595 nm with a UV-Vis (8453 Value System; Agilent Technologies, Santa Clara, CA). The concentration of IR700 was measured by absorption at 689 nm to confirm the number of fluorophore molecules conjugated to each monoclonal antibody molecule. We abbreviate IR700 conjugated to trastuzumab as tra-IR700 and to panitumumab as pan-IR700.

### Optical setup of 3D LC-QPM

The details of the 3D LC-QPM setup were published by T. Yamauchi et al. [14, 15]. Light emitted from a tungsten halogen lamp (center wavelength λc = 800 nm) passes through a Michelson interferometer equipped with two identical water-dipping objective lenses (Nikon CFI Fluor 40XW; Water Immersion, NA = 0.80). The reflected wavefronts from the sample and the reference mirror are projected onto the CCD camera so that they form interference images. Based on the principle of low-coherence interference (also known as “white-light interference”), the interference images can only be observed when the optical-path-length from the beam splitter to the sample and the one to the reference mirror are precisely balanced.

### Observation by 3D LC-QPM

Two hundred thousand cells were seeded on a glass slide to which both an anti-reflection (AR) coating (bandwidth of 550-950 nm) and a reflection enhancement coating were applied [14, 15]. The cells were incubated for 24 hours. 10 μg/mL of tra-IR700 or pan-IR700 were added as appropriate to the culture medium and incubated for 6 hours. After incubation, cells were washed once with phosphate-buffered saline and observed with 3D LC-QPM in 3 mL medium. Baseline data was obtained with a long-pass filter, which eliminates wavelengths less than 780 nm thus preventing NIR-PIT. The cells were then exposed to NIR light (700-950 nm) for four different times without the long-pass filter (48, 64, 76 and over 100 sec) and then the observation was continued with the long-pass filter.

### Image analysis of 3D LC-QPM

All LC-QPM Images were visualized using 3D-rendering software (FluoRender, University of Utah) and analyzed using Image J software. We manually traced the outline of each cell in all sliced sections and measured the cell volume using Image J which was compared to the pre-treated cell. The cell volume was measured by the sum of cross-sectional area of transverse sections at 0.28 μm intervals and calculated the rate of volume increase after NIR-PIT.

### Statistics

Data are expressed as means with standard error (SE). We used a one-way analysis of variance for multiple comparisons followed by Tukey-Kramer. A value of *P* less than 0.05 was considered significant.

## SUPPLEMENTARY MATERIALS FIGURES AND VIDEOS









## Abbreviations

APC: antibody-photoabsorber conjugate
AR: anti-reflection
CTLs: cytotoxic T lymphocytes
DCs: dendritic cells
ICD: immunogenic cell death
IR700: IRDye700DX
NIR-PIT: near infrared photoimmunotherapy
ROS: reactive oxygen species
SE: standard error
sec: seconds
3D LC-QPM: three-dimensional dynamic low-coherent quantitative phase microscopy
