# Supplementary material for: Dynamic changes in the cell membrane on three dimensional low coherent quantitative phase microscopy (3D LC-QPM) after treatment with the near infrared photoimmunotherapy
Source: Oncotarget. 2017 Nov 1;8(61):104295–302. doi: 10.18632/oncotarget.22223 (PMC5732807; doi:10.18632/oncotarget.22223)
Supplement: Supplementary file 1 [file oncotarget-08-104295-s001.pdf]

Dynamic changes in the cell membrane on three dimensional low coherent quantitative phase microscopy (3D LC-QPM) after treatment with the near infrared photoimmunotherapy

SUPPLEMENTARY MATERIALS

Confocal microscopic analysis

3T3/Her cells and MDA-MB468 were each cultured in a cover glass bottomed culture dish and subsequently treated with 10 μg/mL of tra-IR700 or pan-IR700. After PBS washing, fluorescent microscopic imaging was performed using an Olympus IX81 microscope (Olympus America, Inc., Melville, NY) equipped with the following filters (Chroma Technology, Rockingham, VT): excitation wavelength 590–650 nm and emission wavelength range 662.5–737.5 nm. Confocal z-axis stacks obtained from images separated by 0.35-0.5 μm along the z-axis (each exposure time = 500 ms). Three-dimensional reconstruction was performed with the appropriate plugins of Image J. Fluorescence intensity was analyzed with the regions of interest (ROIs), four sites in each z-axis stack, drawn at the cell membrane.

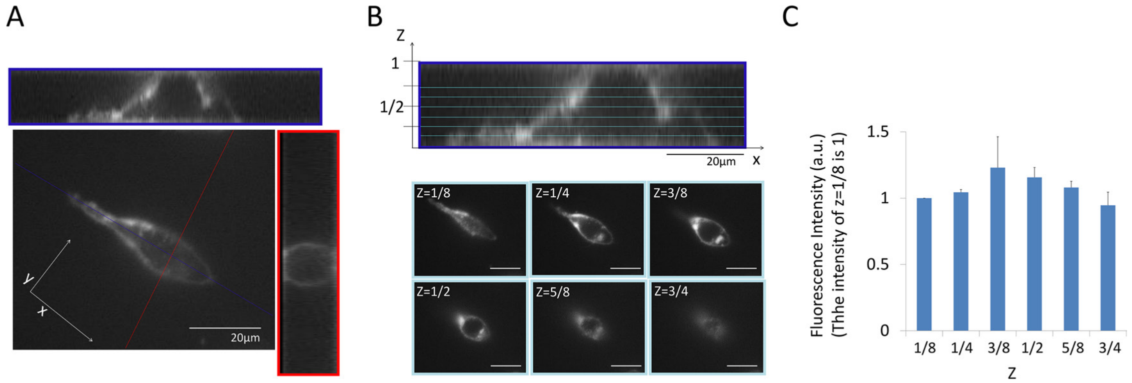

Supplementary Figure 1: Tra-IR700 targeting HER2 homogeneously distributed on the cellular membrane of 3T3/Her cells. Representative images from confocal imaging depict the distribution of HER2 on 3T3/Her cells by three dimensional processing **(A)** and images from separate z axis stacks **(B)**. The highest z value was set as 1. Bars were 20 µm. **(C)** Comparison of the fluorescence intensity of each z-axis stack (z=1/8, 1/4, 3/8, 1/2, 5/8 and 3/4).
Data are means ± SEM. Three cells were analyzed. They were not significant different. a.u., arbitary units

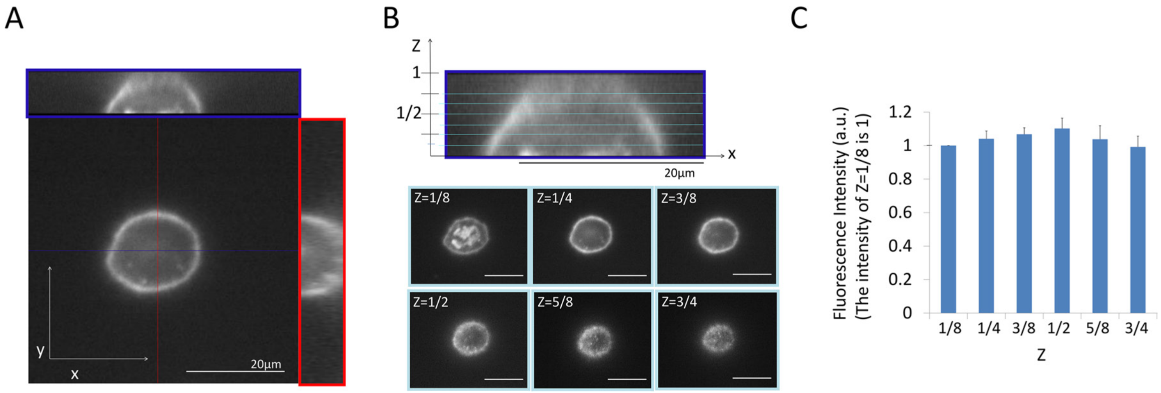

Supplementary Figure 2: Pan-IR700 targeting EGFR also homogeneously distributed on the cellular membrane of MDA-MB468 cells. Representative images from confocal imaging depict the distribution of HER1 on MDA-MB468 cells by three dimensional processing **(A)** and images from separate z axis stacks **(B)**. The highest z value was set as 1. Bars were 20 µm. **(C)** Comparison of the fluorescence intensity of each z-axis stack (z=1/8, 1/4, 3/8, 1/2, 5/8 and 3/4).

Data are means ± SEM. Three cells were analyzed. They were not significant different. a.u., arbitary units

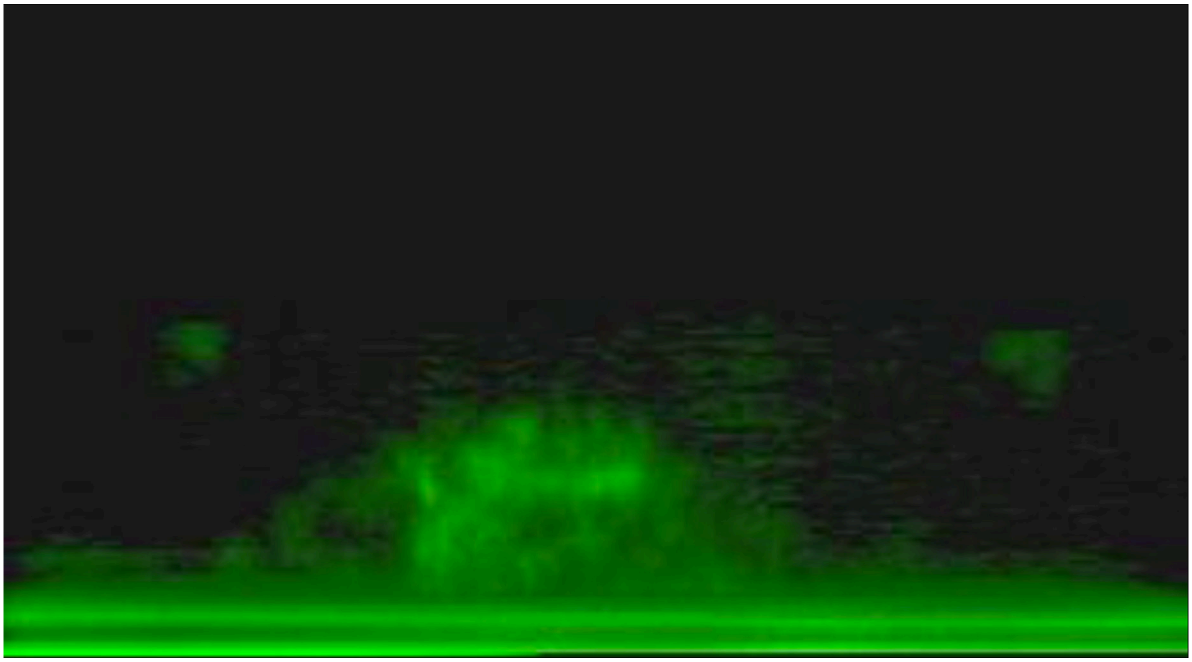

Supplementary Video 1: 3T3 cells increased in volume and ruptured after NIR-PIT. Representative movies of side view from 3D-LC QPM imaging depict morphological dynamics in three regimens of NIR light exposure time, 48 sec **(A)**, 64 sec **(B)** and 76 sec **(C)** shown in Supplementary Movie 1, 2, and 3, respectively. The cell in (A) initially swelled, formed blebs and ruptured. The cell in (B) and the cell in (C) ruptured without antecedent bleb formation and in (C) had already burst by the end of NIR light exposure.

See Supplementary Video 1

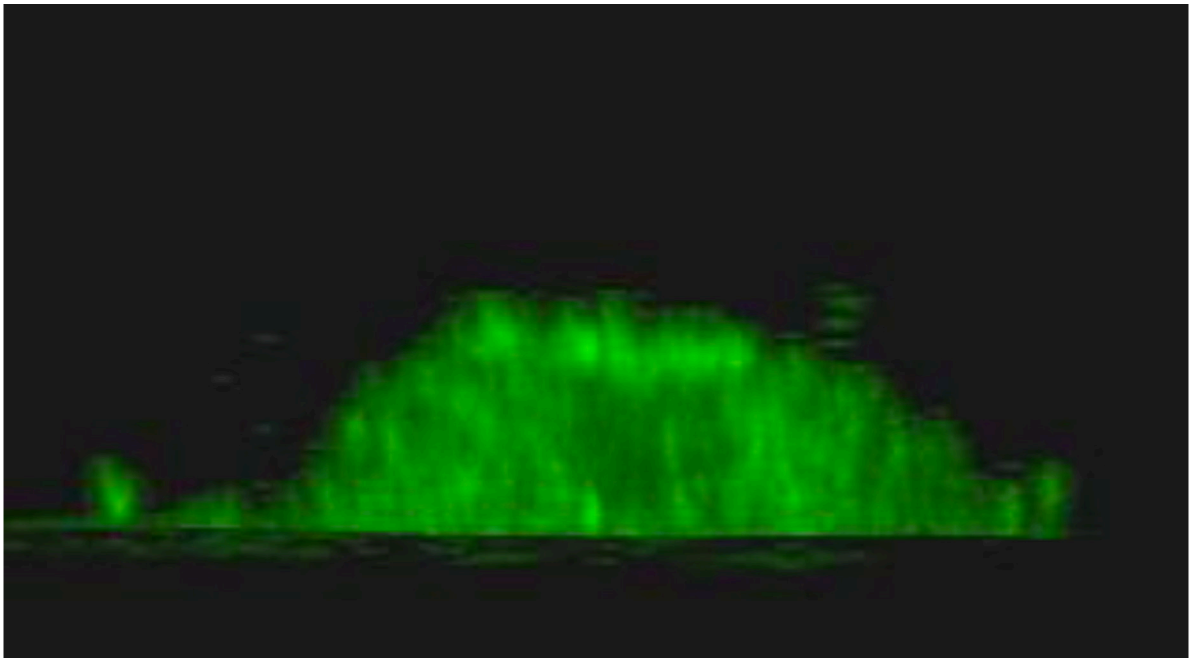

Supplementary Video 2: 3T3 cells increased in volume and ruptured after NIR-PIT. Representative movies of side view from 3D-LC QPM imaging depict morphological dynamics in three regimens of NIR light exposure time, 48 sec **(A)**, 64 sec **(B)** and 76 sec **(C)** shown in Supplementary Movie 1, 2, and 3, respectively. The cell in (A) initially swelled, formed blebs and ruptured. The cell in (B) and the cell in (C) ruptured without antecedent bleb formation and in (C) had already burst by the end of NIR light exposure.

See Supplementary Video 2

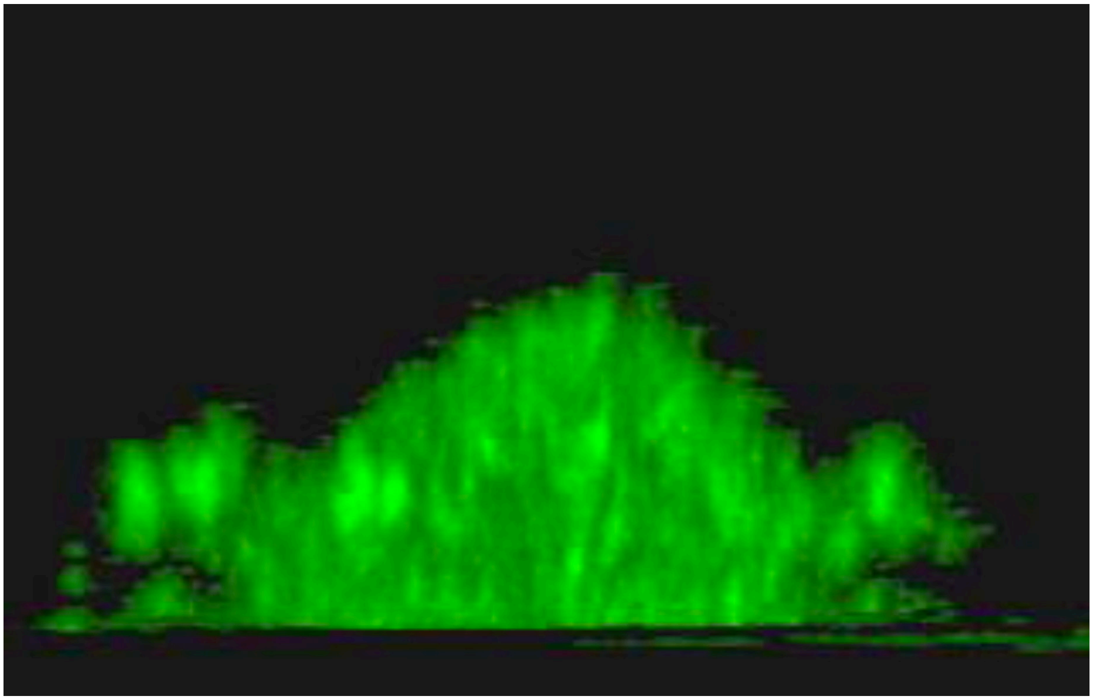

Supplementary Video 3: 3T3 cells increased in volume and ruptured after NIR-PIT. Representative movies of side view from 3D-LC QPM imaging depict morphological dynamics in three regimens of NIR light exposure time, 48 sec **(A)**, 64 sec **(B)** and 76 sec **(C)** shown in Supplementary Movie 1, 2, and 3, respectively. The cell in (A) initially swelled, formed blebs and ruptured. The cell in (B) and the cell in (C) ruptured without antecedent bleb formation and in (C) had already burst by the end of NIR light exposure.

See Supplementary Video 3
